# Supplementary material for: Chromothripsis during telomere crisis is independent of NHEJ, and consistent with a replicative origin
Source: Genome Res. 2019 May;29(5):737–49. doi: 10.1101/gr.240705.118 (PMC6499312; doi:10.1101/gr.240705.118)
Supplement: Supplemental Material [file supp_gr.240705.118_Supplemental_file_1.zip › contigs/annotated_contigs/DB48/contig.2.DB48_length_442_mean_cov_8.47511312217.docx]

**DB48_length_442_mean_cov_8.47511312217**

```
TGAGAACCATGGAAGCCAATGGTGTAGCTCTCAGTCCAAGGCAAAAGCCCTGAGAACCAGGGGGGCTGCTGGTGTGAGTCCTGAAGTCC
 >chr11:124568588-124568790 + E=5e-110
CAAGGCCCAAGAACCAGCACCTCTGGTGTCCAAGGACAGAGAAAAATGGATGTCCCAGCTCCAAAAGAGAATTTGCCCTTCCTCTGCCT

TTTAGTTGGTACTGGTACTT|TTGC|AATGCTAATGGTGCTTCTTGGAGCTATGCAATGCAAAAAGGAGACTCTCCCAAAAGAAAGAGC
                     >chr11:124570068-124570312 + E=2e-135
TTTGGGTGCACCCTTTGATAGCTCAGCTGGTAGAACAGAGGACTATAGGCTGTATAGAAAGAGCTTTGGGTCTAAGAGAGTATGAAAAA

TCATGAGAGAGGGAGAGAGAGAGAGAGAACTTGAACTGTTTTAGAAATATATGCAAAATTTTCTCTGTGGGAAGCTAAGTGTGAGAGT
```
